# *fast*PACE Train-the-Trainer: A scalable new educational program to accelerate training in biomedical innovation, entrepreneurship, and commercialization

**DOI:** 10.1017/cts.2017.306

**Published:** 2018-02-02

**Authors:** Jonathan Servoss, Connie Chang, Jonathan Fay, Kanchan Sehgal Lota, George A. Mashour, Kevin R. Ward

**Affiliations:** 1 Medical School Office of Research, University of Michigan, Ann Arbor, MI, USA; 2 Center for Entrepreneurship, College of Engineering, University of Michigan, Ann Arbor, MI, USA; 3 Michigan Institute for Clinical & Health Research, University of Michigan, Ann Arbor, MI, USA; 4 Department of Anesthesiology, University of Michigan, Ann Arbor, MI, USA; 5 Department of Emergency Medicine, University of Michigan, Ann Arbor, MI, USA

**Keywords:** Innovation, entrepreneurship, commercialization, technology, product development

## Abstract

**Introduction:**

The Institute of Medicine recommended the advance of innovation and entrepreneurship training programs within the Clinical & Translational Science Award (CTSA) program; however, there remains a gap in adoption by CTSA institutes. The University of Michigan’s Michigan Institute for Clinical & Health Research and Fast Forward Medical Innovation (FFMI) partnered to develop a pilot program designed to teach CTSA hubs how to implement innovation and entrepreneurship programs at their home institutions.

**Materials and methods:**

The program provided a 2-day onsite training experience combined with observation of an ongoing course focused on providing biomedical innovation, commercialization and entrepreneurial training to a medical academician audience (FFMI *fast*PACE).

**Results:**

All 9 participating CTSA institutes reported a greater connection to biomedical research commercialization resources. Six launched their own version of the FFMI *fast*PACE course or modified existing programs. Two reported greater collaboration with their technology transfer offices.

**Conclusion:**

The FFMI *fast*PACE course and training program may be suitable for CTSA hubs looking to enhance innovation and entrepreneurship within their institutions and across their innovation ecosystems.

## Introduction

The Clinical & Translational Science Award (CTSA) program was established by the National Institutes of Health (NIH) in 2006 to realize a new vision for translational and clinical science [1]. The University of Michigan (U-M) received a CTSA award in 2007 to fund the Michigan Institute for Clinical and Health Research (MICHR) [2]. In 2011, the NIH developed the National Center for Advancing Translational Science, of which the CTSA program became the centerpiece [3]. At the recommendation of Congress, the NIH engaged the Institute of Medicine, now the National Academy of Medicine (NAM), to evaluate the CTSA program and recommend improvements. The resulting report contained strategies to further enhance the mission of the CTSA program, including a specific recommendation to advance innovation in education and training programs, such as team science, leadership, community engagement, and entrepreneurship [4]. However, there remains a gap in knowledge regarding the optimal interaction of CTSA institutes such as MICHR with the broader innovation and commercialization ecosystems at major research universities.

Academic researchers engaged with the CTSAs have ideas at all stages of development, many of which hold the key to critical breakthroughs in the prevention, diagnosis, and treatment of disease in the form of novel therapeutics, devices, diagnostics, and software/mobile app solutions. However, there is a long, resource intensive route to a viable commercial pathway that leads to a product. Compounding that challenge, the commercialization of research discoveries is often a secondary pursuit for academicians who are time-constrained with clinical care, education, and research responsibilities. In addition, they have competing priorities such as a tenure and promotion goals that often emphasize traditional academic behaviors over other activities.

The NIH has since partnered with The National Science Foundation (NSF) to develop an I-Corps™ program specifically focused on biomedical innovation, leveraging the model of the long-standing and successful program originally designed to improve the impact of recipients of NSF Small Business Innovation Research or Small Business Technology Transfer grants [5]. However, the program is a significant time commitment (7 wk), maintains strict team formation requirements, and only those with a Small Business Innovation Research or Small Business Technology Transfer grants Phase I award are eligible to participate [6]. As a result, these requirements have severely limited the ability for medical academicians with early stage research projects to participate.

In 2012, the U-M Medical School launched a research strategic plan and included development of an innovation and entrepreneurship program: Fast Forward Medical Innovation (FFMI) [7]. FFMI’s mission is to accelerate the transformation of biomedical and life science research ideas into products and services to improve patient care through a unique integration of 3 core sets of activities: (1) *Innovation Navigator* to provide translational funding and mentorship tailored to academic researchers and clinician-scientists, (2) *Business Development* to connect academic researchers with industry partners, and (3) *Innovation, Commercialization, and Entrepreneurship Education* with programs tailored to the busy medical academician (clinician and basic medical scientist), house officer, graduate student, postdoctoral student, and medical student [8]. In response to the NAM report, FFMI partnered with MICHR to develop programs to address the Academy’s recommendations regarding education and training for entrepreneurship, including the *fast*PACE (Program Accelerating Commercialization Education) course, a 4-week experiential learning course customized for the busy medical academician and focused specifically on teaching the foundational concepts for biomedical technology commercialization (and formerly called *The Early Tech Development Course*) [9]. The *fast*PACE course was co-developed with the U-M College of Engineering’s Center for Entrepreneurship, a long-standing NSF I-Corp™ node, to incorporate the core principles and successfully complement the NSF I-Corps™ program [10].

**Fig. 1:**
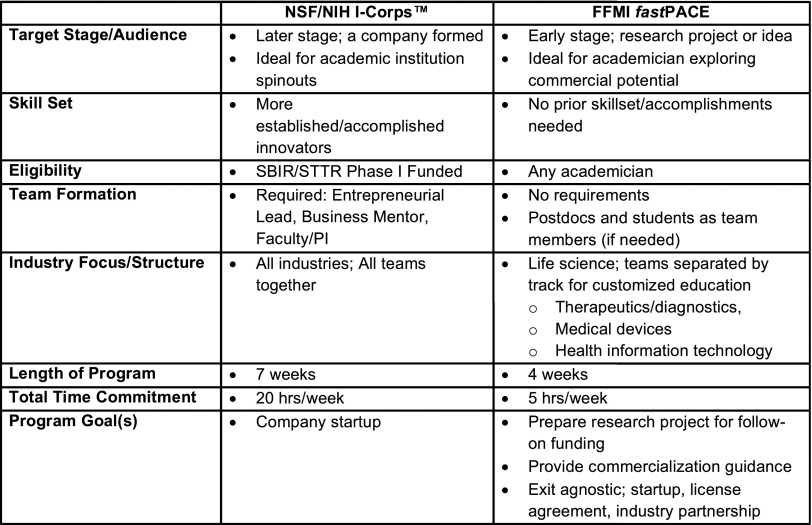
Complementary nature of the Fast Forward Medical Innovation (FFMI) *fast*PACE course to National Science Foundation (NSF)/National Institutes of Health (NIH) I-Corps™. SBIR/STTR, Small Business Innovation Research or Small Business Technology Transfer.

The *fast*PACE course specifically targets *early* stage projects and academicians exploring an exit agnostic route to commercialization in an accelerated timeframe (4 wk). The course does not have eligibility mandates nor team formation requirements and supports enrolled academicians without a strong network of entrepreneurial contacts by connecting them with trained postdocs, medical students, and graduate students interested in a career in biomedical commercialization; as a result, potential barriers keeping the single academician from participating are eliminated. Fig. 1 outlines the complementary nature of the programs and the potential of *fast*PACE to serve as a precursor program to NSF/NIH I-Corps™. Indeed, this potential has been demonstrated by the successful matriculation of three project teams from *fast*PACE cohorts 1–3 into the NSF I-Corps™ program.

Since fall of 2014, the course has successfully completed 6 cohorts, graduated over 250 clinicians, researchers, and students from across the country, and helped 125 project teams navigate the early stages of commercialization by receiving more than $8 million in downstream funding and development partnerships. Outcome metrics from cohorts 1–3 have demonstrated initial success, as 3 project teams have formed startups since completing the course and one has licensed the technology to an existing company. As project teams from cohorts 4–6 (completed as recently as 2017) continue to mature, it is likely that a comparable or greater number of projects will achieve similar outcomes. Importantly, in addition to outcome metrics, the value of the *fast*PACE course extends to the cultivation of an innovation ecosystem that can support the successful execution of such a customized biomedical innovation education program. Key partners for *fast*PACE implementation include the U-M Office of Technology Transfer, core units supporting the research enterprise (eg, clinical trial units, contracts, legal, etc.), diverse schools and colleges (eg, Engineering, Business, Information, etc.), and regional economic development agencies. Members from each group promote and advocate for the course, assist in the recruitment of project teams, and serve as course instructors. The course also serves as a means for recognizing potential gaps in the innovation ecosystem and as a foundation on which to build impactful, collaborative programming to enhance the mission of a CTSA.

### Disseminating *fast*PACE to Other CTSA Institutions and Academic Medical Centers

In recognition of the impact that the *fast*PACE course has had on the acceleration of biomedical research commercialization, as well as its complementarity to NSF/NIH I-Corps™, FFMI and MICHR partnered to execute on the National Center for Advancing Translational Science and CTSA vision to disseminate resources and methodologies that foster innovation in translational science across the CTSA network. In March 2017, FFMI and MICHR launched a pilot *fast*PACE Train-the-Trainer (TtT) program designed to teach CTSA leads and other institutional collaborators how to implement the *fast*PACE course at their home institutions. The program attracted participation from nine CTSA institutions and 16 representatives, providing each with the training, materials, and support to launch their own *fast*PACE program within 6–12 months. Critical success factors, such as key partners and collaboration strategies, were introduced and workshopped to ensure broad campus-wide support and the culture change required for the success of *fas*tPACE. To highlight the approach for disseminating biomedical commercialization education throughout the CTSA network, we fully describe the design, implementation, and outcomes from the pilot *fast*PACE TtT program.

## Materials and Methods

### The fastPACE Course

The *fast*PACE course is tailored to the specific needs and constraints of busy academic researchers and clinicians exploring the commercial potential of biomedical and life science research projects. The course helps learners of all levels develop a basic vocabulary for commercialization and entrepreneurship, and enhances their understanding of key concepts most critical for the commercialization of life science technologies. The desired outcome is to de-risk current and future technologies by preparing academicians for successful commercialization through university exits of all types including entrepreneurship in the form of starting a new company, a license to an existing company or a research agreement with an industry partner.

**Fig. 2:**
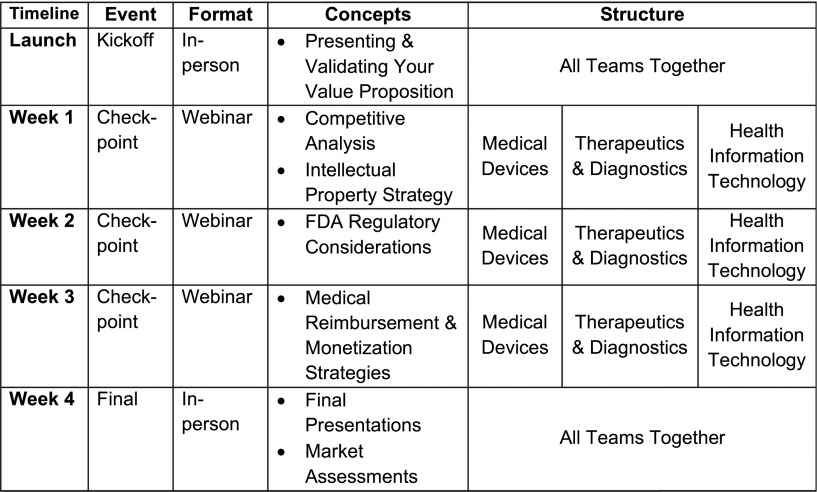
The week-by-week progression of biomedical commercialization concepts and track structure of the *fast*PACE course.

Over 4 weeks, the course covers innovation and entrepreneurship concepts while addressing unique biomedical commercialization risks, such as Food and Drug Administration (FDA) regulations, reimbursement strategies, and intellectual property considerations. By using a track-specific structure, where project teams are streamed into 1 of 3 product verticals (therapeutics/diagnostics, medical devices, and health information technology), each track receives customized education for its unique vertical. For example, the FDA regulatory considerations for a healthcare software/mobile app solution versus a new drug therapy have very different levels of risk, timelines for approvals, and required resources. Fig. 2 provides an overview of the week-by-week progression of biomedical commercialization concepts covered and the track structure of the *fast*PACE course.

### TtT Program Structure

**Fig. 3:**
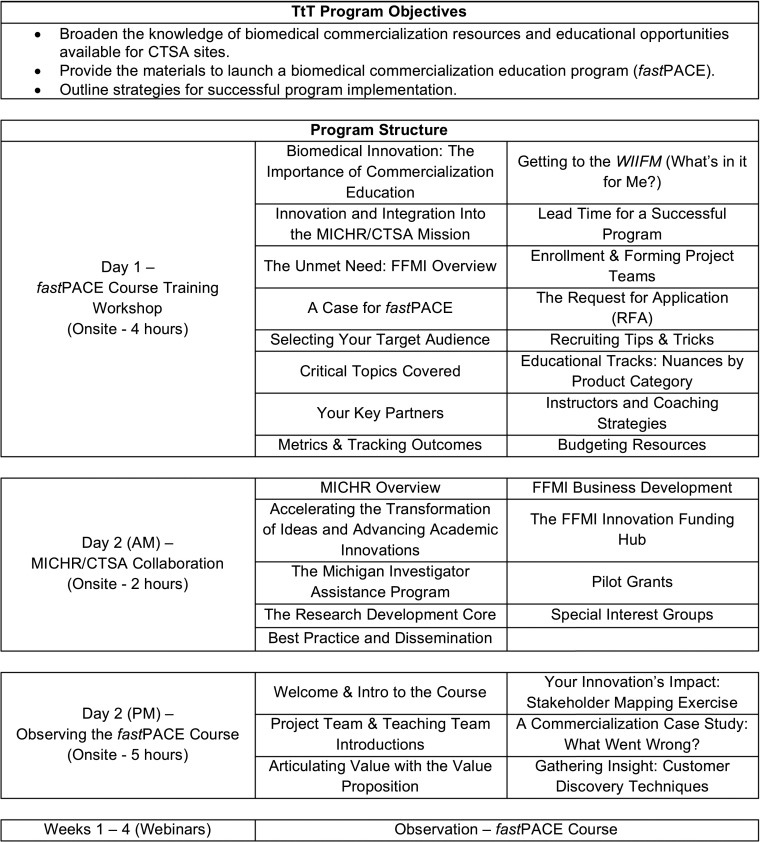
*fast*PACE Train-the-Trainer (TtT) objectives and program structure. CTSA, Clinical & Translational Science Award; MICHR, Michigan Institute for Clinical & Health Research; FFMI, Fast Forward Medical Innovation.

The *fast*PACE TtT program provided a 2-day onsite training experience (at U-M) combined with observation of a *fast*PACE cohort that was launching simultaneously. The 2-day training was divided into 3 parts: (1) a *fast*PACE course training workshop, (2) a MICHR overview and discussion for future CTSA collaboration, and (3) observation of the *fast*PACE course Kickoff Event. Following the 2-day training, program participants observed the remaining weekly progression (4 wk) of the *fast*PACE course via weekly webinars. At the conclusion of the entire program (2-d training and observation), participants were expected to have a broader knowledge of biomedical commercialization education and resources, as well as the materials and strategies to implement the *fast*PACE course at their home institution. Fig. 3 outlines the objectives and full structure of the *fast*PACE TtT program.

### Day 1: *fast*PACE Course Training Workshop

The *fast*PACE course training (day 1) workshop was intended to provide specific details on the design and implementation of the course at U-M. The 4-hour training session addressed key strategies for designing a biomedical commercialization education program at any institution, including collaboration between key campus partners (eg, Office of Technology Transfer, local/regional I-Corps™ program, etc.), the value of expanded promotion and tenure guidelines to include innovation and entrepreneurship metrics, and the value in leveraging student groups across the university to support faculty researchers and clinicians.

Following an introduction by FFMI and MICHR leadership, participants discussed the unmet need for commercialization education at each institution. In a roundtable format, participants shared examples of existing innovation and entrepreneurship programs, as well as any gaps in programming on their campus. Remarkably, none of the existing programs described by the participating institutions specifically targeted biomedical researchers or clinicians, nor did they facilitate collaboration between faculty and students. Participants also shared challenges such as the difficulty of modifying existing programs to meet the specific needs of their CTSA, and navigating the institutional support required to start a new program. Presentations and facilitated discussion by FFMI staff addressed each of these concerns, as well as program planning strategies. For instance, strategies and processes were presented for selecting and targeting an audience of biomedical researchers, clinicians, graduate students, and medical students during a match-making and team formation process. Potential key partners and materials used for support were presented, including innovation and entrepreneurship centers from across the university, and community resource centers with access to local biomedical industry and venture experts. Specific preprogram and postprogram planning activities were also presented in the form of a timeline of key events and dates. Critical activities identified include the assembly of an expert teaching team, the request for applications and project team onboarding, and the program budget. Metrics for tracking success and opportunities for improvement were also discussed with the group. The session concluded with a panel discussion including FFMI, MICHR, and cross-campus collaborating units at U-M, including the Tech Transfer Office (both licensing and venture creation) and the Center for Entrepreneurship at the College of Engineering (NSF I-Corps™ node), to highlight strategies for successful teamwork and to summarize positive outcomes achieved.

### Day 2: MICHR/CTSA Discussion and Collaboration

The 2-hour MICHR/CTSA collaboration session (morning of day 2) was designed to highlight the successful integration and collaboration between MICHR and FFMI, including a portfolio of programs as part of the optional CTSA renewal module for innovation (online Supplementary Appendix) submitted by MICHR in 2016. These included examples of projects utilizing FFMI innovation and entrepreneurship resources and/or MICHR programs as they leveraged various follow-on funding and additional programs to further expedite the translation of the project. Two specific FFMI initiatives presented were Business Development, a dedicated team that focuses on industry research engagement, and the Michigan Translational Research and Commercialization for Life Sciences program, a funding and mentorship program in partnership with the state’s Michigan Economic Development Corporation. Complementary MICHR programs presented included the Michigan Investigator Assistance Program, established to provide IND/IDE consultation and support, the Research Development Core, offering pre-award support and advice for research proposals, and the Pilot Grant program that supports innovative clinical and translational research. The session concluded with a roundtable discussion on best practices for sharing and disseminating materials, training, and additional resources throughout the CTSA network, as well as the discussion of formation of a special interest group focused on commercialization of life science technologies.

### Observing the fastPACE Course

The 2-day onsite training concluded (afternoon of day 2) with the opportunity to observe the course Kickoff Event for a *fast*PACE cohort starting at U-M. By planning the TtT concurrently with a *fast*PACE cohort, participants of the TtT program were able to see the composition of each participating team, the interaction with the assembled teaching team members, and the innovation, entrepreneurship, and biomedical commercialization topics taught throughout the course.

As observing members of the course, TtT participants were provided with the same instructional materials, syllabus, presentation slide decks, notes, and handouts as participating project teams. Specific instruction observed during the course Kickoff Event provided exercises to teach the development and articulation of a value proposition, the mapping of an innovation’s ecosystem of critical stakeholders (to identify gaps in understanding), and the skills in behavior-based interviewing techniques (for eliminating any gaps identified), also known as customer development (discovery), a popular and successfully proven process used during the NSF/NIH I-Corps™ program for launching new ventures [11].

After leaving the 2-day onsite training, TtT participants continued to observe the *fast*PACE course weekly progression via webinar (Fig. 2). Over the course of 4 weeks, participants were able to observe 3 weekly check-points between project teams in the course and the expert teaching team for each track, as well as the final session of the course that featured a final business case presentation by each project team. The 3 check-points allow teaching team members to monitor and discuss the progress made by the project team each week. By observing these check-point webinars, TtT participants were able to see the progress as well, and hear the weekly instruction provided by teaching team members as project teams address certain risks that lead to a success business case. The resulting final business case presentation for each project team addressed the intellectual property, competitive landscape, market size, FDA regulatory process, and monetization strategy across all product categories (device, diagnostic, therapeutic, health IT software/mobile app).

### Implementing the fastPACE Course

At the conclusion of the TtT program, participants were provided full access to a shared folder of slide presentations, links to online videos, and course planning documents for the *fast*PACE course. By sharing these materials, participants were able to download any individual lecture or complete module for the course to be used at their home institution with or without modification. In addition, TtT participants were granted access to the FFMI online learning management system that contained the week-by-week progression of all educational material and course instructions so TtT participants could potentially implement an entirely virtual 4-week course by simply directing their teams to the FFMI Web site. There was no additional cost to participating institutions for access to these materials; however, institutions were simply asked to partner by co-branding the slides and providing attribution to the U-M’s originating program.

The *fast*PACE course is easily customizable to the institution and its needs based on the target audience, which may range from faculty to graduate students. The *fast*PACE course could be developed as a total online course but, in our opinion, provisions should be made for robust implementation of the webinar check-points and critical closing experience of presenting the final business case in a live setting. The total cost for implementing a *fast*PACE course without the TtT experience and the supportive materials from FFMI is approximately $24,000 [9]. The majority of these expenses are instructor stipends and administrative oversight for the course. Participants in the TtT program are provided the *fast*PACE educational content, online platform for delivery, and additional materials so the direct costs for implementing the course are considerably less. It was suggested during the TtT program that funding to implement the *fast*PACE course could occur through internal budget reallocations, cost sharing with medical and other schools or colleges participating, and allocating for such in future renewals or initial grant submissions.

### Participating Institutions and Participants

CTSA executives and administrative staff were targeted to participate in the TtT program. To begin recruitment efforts, communications were sent via email to CTSA directors ~4 months prior to the start of the program. The email communications detailed the objectives and potential benefits to participating CTSAs. Furthermore, members of the Midwest Area Research Consortium for Health (a regional CTSA network) were invited to attend an informational webinar detailing the program agenda 1 month prior to the start. Communications were also sent to individual CTSA working groups, including the Clinical Research Training for Investigators Workgroup that hosted a 15-minute informational presentation during a scheduled conference call. Additional recruitment efforts in the form of follow-up email communication and phone conversations were critical in assembling the final roster CTSA institutions and participants.

**Fig. 4:**
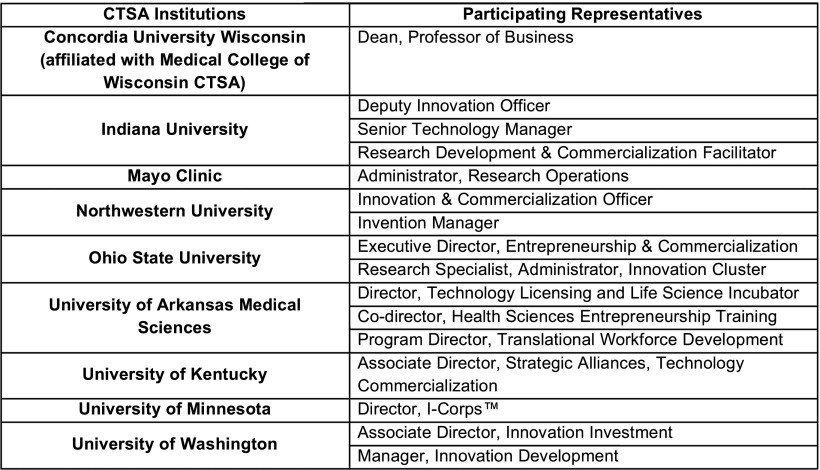
Participating Clinical & Translational Science Award (CTSA) institutions and representatives by title.

In total, 9 CTSA institutions and 16 representatives participated in the program. Each individual institution determined what units, functions, and representative(s) should participate in the training program, considering who would be best equipped to implement the *fast*PACE program at their home institution. Fig. 4 lists the participating CTSA institutions and titles of each TtT participant.

### Program Expenses

Total direct expenses for FFMI and MICHR to provide the TtT program were approximately $4800 (indirect or administrative/overhead expenses not included). Hosting for 16 institutional guests to attend the 2-day onsite training totaled $3400. Remaining expenses were $1400 for the design, printing, and distribution of all training materials including training binders, presentations, handouts, and other materials to allow institutional representatives to implement the FFMI *fast*PACE course. There was no registration fee for the participating institutions; however, each institutional representative provided their own travel and accommodations estimated at $500 each.

## Results

### 2-Day Onsite Training Evaluation

An evaluation was administered via online survey tool (Qualtrics™) immediately following the initial 2-day onsite training to evaluate the program’s effectiveness across a variety of criteria, including broadening the participants’ knowledge base of biomedical commercialization resources and education, assessing potential impact on participating institutions by implementing all or part of the FFMI *fast*PACE course, and interest in continued collaboration. The evaluation received a 31% response rate of the 16 participants (n=5). Following the onsite training, all 5 respondents strongly agreed or agreed that the program met their expectations, it was delivered appropriately across 2 days, and it successfully broadened their knowledge of biomedical commercialization resources and education. In addition, all 5 respondents strongly agreed or agreed that the program will directly impact their institution because they plan to implement all or part of the FFMI *fast*PACE course.

### Preliminary Outcomes from the Full TtT Program

Phone interviews were conducted with all TtT participants 3 months postprogram to capture preliminary outcomes, in the form of implementation activities and impact from attending and observing the entire 4-week program. Six of the 9 attending institutions, including University of Arkansas Medical Sciences, Ohio State University, Northwestern University, University of Kentucky, University of Washington, and Indiana University were using shared materials and online resources made available during the TtT program to launch their own version of the FFMI *fast*PACE course or to modify and bolster existing innovation and entrepreneurship programs at their institution. Two of the 9 institutions have recommended that project teams from their institution enroll in a *fast*PACE course cohort at U-M prior to receiving funding from their own translational science programs. As a near-term and temporary approach, this will allow teams to access *fast*PACE training while their home institution works toward potential implementation of its own program. Two participating representatives reported resulting collaboration between the technology transfer office and CTSA at their home institution. Perhaps most importantly, representatives from all attending institutions reported a greater connection to biomedical research commercialization resources and the desire to stay connected through a potential new CTSA-based consortium focused on biomedical research, innovation, and commercialization.

## Discussion

Based on analysis of the evaluation data and preliminary outcomes, the *fast*PACE TtT program successfully disseminated a valuable biomedical commercialization education program to the group of participating CTSA institutions. This result represents a proof of principle that CTSA institutes and institutional commercialization and entrepreneurship programs can partner, both internally and externally, to address the NAM’s recommendation to the CTSA program regarding enhancing its focus on innovation and entrepreneurship in education and training programs.

In addition, the *fast*PACE TtT program successfully addressed strategies for each participating institution to identify existing biomedical commercialization resources within its own ecosystem to enhance their impact through partnerships via collaborative programs such as *fast*PACE. As previously mentioned, none of the participating institutions knew of existing programs that addressed the unique and specific needs of biomedical faculty and academic researchers who are time-constrained and also require tailored education on specific concepts that are most relevant to the successful commercialization of their technologies. Interestingly, some of the participating institutions were in relatively close proximity to existing I-Corps™ programs, further underscoring the opportunity for *fast*PACE to complement I-Corps™ in preparing teams at an earlier stage than those that would typically enroll.

The success of *fast*PACE at U-M has led to benefits that further address the NAM’s critique and uncovered new opportunities for partnering with our CTSA organization (MICHR), as well as many others. For example, FFMI and MICHR have partnered to create the nation’s first Continuing Medical Education accredited series of seminars for providers in an academic medical center that focuses on core topics related to commercialization, industry partnership, and entrepreneurship. Furthermore, FFMI and MICHR are working together to enhance workforce readiness with an internship program that places postdoctoral fellows and graduate students in industry settings. Students seeking a career in industry will participate in commercialization courses, including *fast*PACE, as a prerequisite to obtain foundational training and placement preparedness. In addition to *fast*PACE, FFMI has launched other commercialization education programs within the PACE portfolio with tailored formats for multiple audiences. The *full*PACE program, a 12-week multidisciplinary team-based program for medical residents, fellows, graduate students, and postdocs has graduated 2 cohorts of ~35 participants since 2015. Beginning in the fall of 2017, the *full*PACE program will be offered through the U-M Rackham Graduate School as a 3-credit hour course. The *first*PACE series, a 3-part series on medical needs finding, design thinking for healthcare innovations, and value proposition analysis is delivered to medical students in a novel co-curricular program, known as the Path of Excellence in innovation, entrepreneurship, and commercialization, offered in collaboration with the U-M Medical School. Finally, *fitted*PACE offerings are customized learning opportunities for sponsoring departments within the U-M Medical School to help achieve specific strategic objectives related to innovation. As a customized program, *fitted*PACE has offered everything from a 9-month experiential program for an entire department of 90 faculty to short ideation sessions for an individual product vertical (therapeutics, devices, diagnostics, and health information technology) that enhance learning and generate new ideas. It is clear that the potential value of a program such as *fast*PACE extends well beyond the parameters of a single course or framework and can have a far greater impact across the entire innovation ecosystem in which it is implemented.

## Conclusion

In conclusion, the partnership between FFMI and MICHR presents a novel model and opportunity to deliver on the overall CTSA mission to disseminate best practices and successful methodologies that accelerate the process of translating laboratory discoveries into clinical practice. Preliminary experience with the program suggests that the *fast*PACE course and its TtT program may be suitable for a broad range of academic medical centers, including those with CTSA programs looking to enhance opportunities for innovation and entrepreneurship within their ecosystems.

## Supplementary material

For supplementary material accompanying this paper visit https://doi.org/10.1017/cts.2017.306. click here to view supplementary material
