# Supplementary material for: fastPACE Train-the-Trainer: A scalable new educational program to accelerate training in biomedical innovation, entrepreneurship, and commercialization
Source: J Clin Transl Sci. 2018 Feb 2;1(5):271–7. doi: 10.1017/cts.2017.306 (PMC5915817; doi:10.1017/cts.2017.306)
Supplement: Supplementary file 1 [file S2059866117003065sup001.docx]

**APPENDIX**

**MICHR Optional CTSA Module H: Innovation, Commercialization, and Entrepreneurship**

**Specific Aims**

Launched in 2014, the University of Michigan (U-M) Fast Forward Medical Innovation (FFMI) program accelerates the transformation of ideas to improve clinical care into a reality. FFMI stands unique among its peers in the integration of three key strategies: 1) Innovation Navigator, 2) Business Development, and 3) Innovation, Commercialization, and Entrepreneurship Education. MICHR was critical in the creation of FFMI and continues to be a strong partner in advancing the development of biomedical discoveries along the translational continuum. After almost two years in operation, FFMI has been fully developed and has demonstrated success in encouraging technology transfer and commercialization in order to bring academic innovations to the marketplace. We now turn to dissemination at the local, regional, and national levels. By accomplishing the following aims, MICHR and FFMI will create an integrated, comprehensive approach to increase the potential for more academic discoveries to be translated into interventions that improve health:

***Specific Aim 1: Scale the Innovation Navigator Program to the entire U-M health sciences community, providing milestone-driven innovation, commercialization funding, and expert mentorship.***

Rationale: FFMI offers consultation for multistage technology development, funding, and mentorship (i.e., the Innovation Navigator Program) for successful commercialization of biomedical discoveries, but currently its offerings are limited to the Medical School.

Approach: MICHR, as a U-M institute with a university-wide reach, will enable FFMI to extend the Innovation Navigator Program that supports innovation and commercialization to the broader U-M community engaged in health science innovation. In so doing, we will empower U-M research teams with the knowledge, skills, and support necessary to accelerate the translation of their research to the clinic.

***Specific Aim 2: Provide new externship opportunities that offer real-world experiences with partners, including biomedical companies, regulatory and intellectual property consultancies, and technology transfer organizations.***

Rationale: The successful translational scientist-in-training must develop not only scientific skills but also build skills in communication to non-scientific audiences, leadership, effective functioning in a team, and a fluency in the commercialization process.

Approach: MICHR endeavors to provide scholars and trainees with practical, experiential learning that allows them to obtain a working knowledge of the product development pathway and commercialization process as critical components of translating new discoveries as well as exposure to career development opportunities. To that end, MICHR will engage U-M campus partners, especially FFMI and the U-M Business Engagement Center, to join forces to develop competitive externship opportunities with both established and start-up biomedical companies, allowing trainees to learn first-hand about product development, expectations for a career in industry, and business drivers for academic-industry partnerships.

***Specific Aim 3: Disseminate innovation, commercialization, and entrepreneurial education programs across the State of Michigan.***

Rationale: A significant challenge facing academic health science researchers who aspire to move technologies from idea to impact is the lack of opportunity to learn about the innovation, commercialization, and entrepreneurship process. Compounding this challenge are the growing demands on academicians to be more productive in their clinical, teaching, and research duties with increasingly constrained resources. These factors make it critical to provide high-yield, in-depth curricula in biomedical commercialization that are tailored to the busy scientist or clinician.

Approach: MICHR will partner with FFMI to create a statewide innovation network to disseminate commercialization and entrepreneurial education programs to accelerate discovery and the development of biomedical innovations.

Together, these programs will prepare U-M clinical-translational researchers at all levels–students, post-doctoral fellows, and faculty–to translate more effectively their promising discoveries. Our long-term goal is to improve patient care and enhance human health through biomedical innovations emanating from U-M, across the State, and beyond.

**Significance**

With 422 invention disclosures and 160 issued patents in 2015, U-M has tremendous potential to create a positive impact on human health and society. To facilitate the translation of discoveries to impact, the Fast Forward Medical Innovation (FFMI) program was established to accelerate the transformation of biomedical innovations into patient and health impact. MICHR has played a foundational role in conceptualizing, establishing, and sustaining the FFMI program. While the number of academic medical center innovation programs around the nation is growing, FFMI remains unique in its integration of 1) product development funding and mentorship through an “Innovation Navigator” Program, 2) industry engagement via a business development team, and 3) innovation and commercialization education. The combination of these strategically integrated programs enables FFMI to build and nurture a culture supportive of biomedical innovation with the goal of accelerating new products coming to market as well as to catalyze clinical-translational scientists to think differently about their research. During the past two years, FFMI has already interacted with nearly 250 faculty through its technology mining function, received and triaged >100 submissions for proof-of-concept funding, and received 75 submissions for commercialization funding and mentorship, with 35 projects awarded. As this platform was established to de-risk early-stage projects for technology development, it was imperative to also de-risk the potential impact on a faculty’s academic career as they embarked on entrepreneurial activities. To that end, FFMI spearheaded the modernization of the Medical School’s promotion and tenure guidelines to value and reward innovation and entrepreneurship, thereby creating a culture shift to make these activities an expected and natural academic behavior. Together, MICHR and FFMI will create an integrated, comprehensive infrastructure to increase the potential for more academic discoveries reaching the marketplace and having impact on patients.

**Investigators**

The Executive Director for FFMI, Kevin Ward, MD, will provide leadership that will fully leverage the wealth of resources of MICHR, other U-M units, and key partners across the State of Michigan to achieve efficiencies of scope and scale. Dr. Ward is an emergency medicine physician as well as prolific inventor and product developer in the field of critical care, with numerous patient and industry partnerships to develop new technologies across diverse domains from battlefield medicine to civilian intensive care units. Connie Chang, MBA, Managing Director of FFMI, has extensive outside industry, business and commercialization experience in large pharmaceutical and biotechnology companies. FFMI’s Commercialization Program Director, Bradley Martin, PhD, brings to the team expertise in large pharmaceutical Research & Development (R&D), small biotechnology company R&D, and commercialization mentorship and education.

**Innovation**

MICHR and FFMI will scale education programs to act as a force multiplier for innovation and entrepreneurship. Our work is innovative in that it leverages the Clinical Translational Science Award (CTSA) to create a commercialization engine for academic discoveries reaching real impact on the health of our patients and communities by leveraging infrastructure across U-M and the State of Michigan. Most biomedical scientists have not had exposure to and training on navigating the commercialization pathway. Traditionally scientific training has focused on teaching the basic knowledge, methodologies, and best practices of a specific scientific discipline. To accelerate the pace and increase the efficiency of translating academic innovations to preventive, diagnostic, and therapeutic interventions, we address this training gap. Furthermore, through this work we will innovate new methods and approaches to commercial translation of academic discoveries that can be broadly shared and adopted across the CTSA network and beyond.

**Approach**

***Specific Aim 1: Scale the Innovation Navigator Program to the U-M health sciences community, providing milestone-driven innovation, commercialization funding, and expert mentorship.***

**Introduction:** In the proposed funding period, MICHR and FFMI will leverage their synergistic resources and more fully integrate their activities related to innovation, commercialization, and entrepreneurship. Since its inception two years ago, FFMI has focused primarily on faculty-related programs within the U-M Medical School. An enhanced partnership with MICHR will broaden and extend existing education, funding, and mentorship programs to other schools and colleges at U-M engaged in health science innovation. Commercialization and entrepreneurship education programs are described in **D. Translational Endeavors** and **Aim 3** below. This aim complements the educational offerings with technology development funding and expert mentorship to equip clinical-translational researchers at U-M with the tools and support necessary to successfully engage in the commercialization process to translate their discoveries.

**Methodology:** Many nascent technologies die in “the valley of death” between traditional funding sponsors focused on basic research and venture investors seeking late-stage technologies. FFMI has developed a series of efforts, termed the Innovation Navigator Program, which are intended to address barriers in the commercialization process and speed the pace of translation. The goal of the Innovation Navigator Program is to spur innovation from U-M translational scientists and support projects of high commercial potential to successfully traverse translational gaps. Specifically, these efforts include:

- **Identifying technologies with high clinical and commercial value** via proactive technology mining, “idea” consultation, and establishing a front door for biomedical innovation.
- **Funding translational research activities** of those projects determined to be on a viable path toward commercialization. All work is milestone driven and directed towards a near-term (12-18 month) university exit, including licenses to existing companies and/or start-up formation.
- **Mentoring to progress projects**; we have established a network of industry professionals with real-world experience in technology development and commercialization to provide mentorship and expertise to investigators.

These elements of the Innovation Navigator Program will nurture innovation and de-risk technologies, creating a pipeline of U-M discoveries with an increased likelihood of advancing along the clinical commercialization pathway and that are better positioned for further development by industry. One of the primary difficulties academic investigators encounter in translating promising research findings to the clinic is a lack of funding—and guidance—to design and execute the safety and toxicology studies required to secure an Investigational New Drug (IND) or Investigational Device Exemption (IDE) application approval from the Food and Drug Administration and to conduct first-in-human studies. Traditional extramural funding sources are unlikely to fund such work, and the investment community is hesitant to invest in high-risk, preclinical technologies—leaving innovators with limited options for translating their technologies. MICHR is uniquely positioned to address these critical needs by providing: 1) faculty with funding to conduct safety studies; 2) regulatory guidance; 3) assistance in assembling the data package; 4) supporting IND/IDE application preparation, submission, and maintenance; 5) biostatistic and study design support; and 5) ultimately, execution of first-in-human clinical trials. These important services significantly enrich the Innovation Navigator Program.

MICHR and FFMI will leverage their synergistic assets and partner to extend the Innovation Navigator Program to the broader U-M community engaged in health science innovation. The Innovation Navigator Program will offer consultative services, innovation funding, and mentoring at all stages of technology development to faculty, students, and post-doctoral fellows from the U-M biomedical research community. Innovators can access resources by first having an “idea” consultation with an expert member(s) of the Innovation Navigator team. The program offers early- (Kickstart), mid- (MTRAC), and late-stage (Monroe-Brown) funding mechanisms that enable the continued development and progression of technologies toward clinical applications (**Table H.1**).

| ***Table H1. Technology Development Funding Mechanisms (Institutionally Funded)*** | | |
| --- | --- | --- |
| Program | Purpose | Characteristics |
| **Kickstart** | Proof of concept/prototyping funding program to help accelerate ideas through to a scientific feasibility stage; informs a “go/no go” decision on further technology development. | - Rolling submission - $25-50K in funding - 6-month “go/no go” decision |
| **Michigan Translational Research and Commercialization (MTRAC) for Life Sciences** | Translational fund for mid-stage technologies. Funded projects have been determined to have a high commercial value and are 18-24 months from commercialization. This mechanism receives support from the State of Michigan. | - Annual funding cycle - $150-250K in funding - External Oversight Committee - Milestone driven - Experienced project management |
| **Monroe-Brown**  **Pre-Seed Fund** | Funding mechanism from philanthropic donor to make pre-valuation investments in nascent biomedical start-ups emanating from technologies jointly created by Engineering and Medical faculty members. | - Equity investments in start-ups - Initial investments of up to $250K - External Investment Committee |

Importantly, expert mentorship accompanies each stage of funding, offering investigators unique domain expertise. For example, the Michigan Translational Research and Commercialization (MTRAC) for Life Sciences mechanism has an External Oversight Committee comprised of industry leaders, patent attorneys, venture capitalists, and translational scientists who review the applications and select projects with the greatest commercial potential for funding. The External Oversight Committee also provides hands-on mentorship, assisting investigators with technical, regulatory, business, legal, and university issues to shepherd biomedical innovations along the commercialization path. In addition, we have identified U-M faculty champions who have extensive and highly relevant commercialization and entrepreneurial experience as serial innovators and entrepreneurs with industry-licensed technologies and/or venture- and industry-backed companies. The faculty champions offer additional mentorship. Funded projects will also benefit from experienced project management to help investigators with milestones, timelines, and management of critical-path activities. Lastly, the Innovation Navigator team has a strong, collaborative partnership with the U-M Office of Technology Transfer, who will assist with submission of invention disclosures, market analysis, intellectual property strategies, commercialization decision points, business formation, and licensing agreement strategies.

**Metrics & Expected Outcomes:** MICHR will ensure timely data and metrics collection. We will track the number of idea consultations conducted, new invention disclosures submitted (including new and repeat inventors), publications, licenses/options, and start-up companies. We also will track funding through the expanded university-wide Kickstart and MTRAC programs, measuring project-specific, follow-on funding and other commercialization outcomes from these mechanisms. Integration of MICHR and FFMI expertise and resources through the Innovation Navigator Program will create a more comprehensive and robust commercialization pathway with coordinated consultation, mentorship, regulatory assessment and support, milestone development, study design and analysis, and funding. We expect to further catalyze translation of health science technologies into treatments through the projects we support. In addition, we expect to develop and disseminate new methods and processes that improve the innovation, commercialization, and entrepreneurship process that can be adopted at other academic medical centers.

**Potential Problems & Alternative Strategies:** Given MICHR’s extensive experience offering campus-wide educational programs and FFMI’s early successes in the Medical School, we are confident that we can scale the Innovation Navigator Program to the other health science schools. To ensure its success, however, we will identify and engage faculty champions that are locally regarded within the schools and colleges. We will also leverage MICHR’s extensive network of clinical-translational investigators to create momentum. MICHR and FFMI will also work with other schools’ and colleges’ promotion and tenure committees, if needed, to ensure entrepreneurial activities are rewarded.

***Specific Aim 2: Provide new externship opportunities that offer real-world experiences with partners, including biomedical companies, regulatory and intellectual property consultancies, and technology transfer organizations.***

**Introduction:** Synergies between the public and private sectors as it relates to health science innovation and commercialization of new technologies cannot be underestimated. Academia brings the power of basic and translational research, including the understanding of the biological pathways, mechanisms, and targets that underpin biomedical technology development, coupled with access to patient populations for clinical trials. Industry brings their massive target libraries, manufacturing capabilities, and other R&D tools to accelerate the development of discoveries toward a future product. However, collaboration between academic researchers and industry can be complicated by the fact that academic researchers are best equipped to engage in scientific discussions, while a successful industry-academic collaboration must include not only scientific idea exchange but also business discussions related to the scope of work, budget, confidentiality, expectations for publications, intellectual property terms, and conflicts of interest, to name a few. The ability of a university engaged in translational research to partner effectively with external private sector entities is a critical success factor in accelerating innovations to patient care, thus it is critical to train the translational workforce of tomorrow to be fully prepared to navigate and thrive in this environment.

**Methodology:** Recognizing the need to support research faculty engaged in industry collaborations, U-M has developed two functions specifically for industry relations: the FFMI Business Development team, a group of business professionals with biomedical industry experience based in the Medical School, and the U-M Business Engagement Center (BEC), a central campus group tasked with corporate relations across multiple industry sectors for the entire university. As a result, U-M has experienced growth in co-development agreements, Master Alliance Agreements, pre-competitive consortia, and other approaches for multi-year/multi-investigator collaborations with industry. MICHR will engage campus partners, especially the Business Development team and the Business Engagement Center, to offer robust externship opportunities and other learning avenues to prepare students, post-doctoral fellows, and research faculty for commercial translation of promising research, industry collaborations, and potential careers in the private sector.

*Industry Externship:* In this funding period, MICHR will work with our university colleagues to establish externship opportunities with private sector partners, especially with those entities that we have established research collaborations and relationships. Placement of scholars and trainees into externships will be a highly competitive process, with a successfully-completed internship in commercialization and entrepreneurship (described in **Section D. Translational Endeavors**) as a prerequisite to this structured externship. MICHR’s Education and Mentoring Group has a robust process in place for the recruitment and selection of trainees and scholars that will be employed for the selection of the externs. We anticipate 2-3 scholars or trainees annually will be selected for these highly desirable externship opportunities. The externships, approximately 8-12 weeks in length, will provide the extern with the opportunity to work within the industry partner’s organization on a discrete project. The extern will fully engage in working toward the industry partner’s business objectives; developing direct experience in product development, regulatory affairs, and commercialization; learning about corporate functions; and being part of the organization’s team. Working with business and scientific leaders throughout the company will allow the extern to develop a corporate network and establish potential career development mentors. MICHR will provide a stipend for the duration of the experience. Such externship opportunities will provide trainees with broader, real-world perspectives to inform their own approach to translational research as well as offer career exploration.

MICHR and FFMI will co-launch additional targeted offerings to provide insight to and create opportunities for clinical and translational researchers at U-M to engage with industry:

*Industry Innovation Workshops:* We will develop new workshops specific to learning about how to engage in industry-academic collaborative research and considerations for a career in industry. These learning modules will include lectures where faculty, scholars, and trainees can explore these concepts in greater detail with guest experts from industry. Live webinars will supplement the content delivered during the event with the added convenience of allowing MICHR to archive the programs which will then be included in our online repository of learning modules as well as used in other course offerings in MICHR’s and FFMI’s education curricula. Lastly, interactive panels featuring industry and academic experts will be hosted to provide an opportunity for learners to hear from multiple perspectives about the intricacies of academic-industry partnerships as well as career paths in industry.

*Industry Ideation Sessions:* Interactive, theme-based ideation sessions will be structured to bring industry partners together with academic researchers and students to brainstorm key areas related to industry and academic partnerships and interactions. FFMI has experience in hosting multiple ideation events using the “un-conference” approach where attendees drive the discussion and agenda and, ultimately, identify and drive new ideas, projects, and approaches to accelerating industry-academic collaborations.

*Industry Networking Travel Grants:* MICHR will establish a small pool of its institutional funds to award post-doctoral fellows and students opportunities to participate in major biotech conferences in partnership with FFMI. For instance, the FFMI Business Development team sends a small group each year to the annual International Convention of the Biotechnology Innovation Organization (BIO). BIO represents more than 1,100 biotechnology companies, academic institutions, state biotechnology centers, and related organizations from across the globe. The BIO convention offers attendees the opportunity to learn about the research and development of innovative healthcare and biotechnology products, network with other attendees, and hear about trends in the biotechnology industry.

**Metrics & Expected Outcomes:** Activity-based metrics will include tracking the number and demographics of applicants, offers, and appointments for externship opportunities as well as using semi-structured interviews to assess the experience and satisfaction of the trainee and partner. We also will track longer-term research and career progression for each extern. We expect that the externship will inform the trainees approach to their research and pursuit of translational activities as well as spurring productive industry collaborations.

**Potential Problems & Alternative Strategies:** Developing high-quality, productive externship programs can be challenging and requires clear definition of expectations and significant, ongoing relationship management with partners. Recognizing the value of real-world, experiential learning opportunities for our trainees, MICHR is committed to devoting the time and effort it will take to foster strong relationships to make the experience positive and productive for our trainees.

***Specific Aim 3: Disseminate innovation, commercialization, and entrepreneurial education programs across the State of Michigan.***

**Introduction:** MICHR, in partnership with FFMI, will create an innovation network and commercialization engine to accelerate the discovery and development of biomedical innovations from across the State of Michigan. MICHR is well positioned to be the hub to drive biomedical innovation across our region by leveraging existing resources and relationships from across the state through MICHR’s Community Engagement Program (**C. Community and Collaboration**), FFMI, and the U-M Business Engagement Center. As a directive of the National Center for Advancing Translational Science, CTSA hubs “*are to coordinate and collaborate with multiple spokes”* and “*disseminate innovations and solutions to the translational research community as a whole.*” To that end, creating an innovation network with spokes targeted to key areas across the State of Michigan establishes a robust infrastructure for deploying additional clinical-translational initiatives and best practices statewide, thereby amplifying the role of MICHR as a CTSA hub regionally.

With partnerships across the State of Michigan, MICHR and leverage FFMI to build upon the foundation of biomedical innovation at U-M to create scalable programs and processes to identify, incubate, and accelerate technologies from participating institutions across the innovation network. The potential for biomedical technology commercialization in the State of Michigan is significant (**Table H2**). Based on fiscal year 2013 data for Michigan State University (MSU), Wayne State University (WSU), the Upper Peninsula’s Michigan Technological University (MTU), and U-M, the annual number of invention disclosures totals 662. While these technologies are not all biomedical at their inception, there are tremendous synergies and potential for impacting healthcare through collaboration. For instance, MTU brings deep expertise in engineering and advanced materials research with potential biomedical applications. MSU brings extensive human and animal health research expertise, preclinical/animal model systems as well as agriscience expertise that could be directed toward biomedical solutions. Detroit is home to WSU, a thriving urban university adjacent to a vibrant and growing large medical district (Detroit Medical Center). Additional relationships on the west side of the State include health systems and research institutes that are additional drivers of biomedical innovation.

| *Table H2. Technology Transfer Metrics for Select Universities in the State of Michigan* | | | | | |
| --- | --- | --- | --- | --- | --- |
| FY 2013 | Invention Disclosures | Patent Applications Filed | Patents Issued | Licenses &  Options Executed | Start-ups Formed |
| U-M | 421 | 148 | 128 | 108 | 9 |
| Michigan State | 122 | 49 | 46 | 33 | 1 |
| Wayne State | 68 | 30 | 10 | 3 | 0 |
| Michigan Technological | 51 | 10 | 4 | 13 | 2 |
| Total | 662 | 237 | 188 | 157 | 12 |

**Methodology:** MICHR is well positioned to accelerate the translation and dissemination of scientific discoveries to market. MICHR has supported and leveraged the educational programs offered by FFMI. During the past two years, FFMI has offered its portfolio of innovation, commercialization, and entrepreneurship education programs to >1200 participants. FFMI’s current educational offerings include an accredited *AMA PRA Category 1 Credit™* (CME) lecture series, supplemental webinars, an online library of enduring materials, product themed innovation and ideation sessions, and an experiential Early Technology Development (ETD) course, which is an accelerated biomedical commercialization course modeled on the National Science Foundation’s I-Corps™ program and co-developed with the U-M College of Engineering’s Center for Entrepreneurship, an NSF I-Corps™ node. Importantly, the ETD course has been *modified to meet the needs of the busy physician scientist and biomedical academician* looking to explore the commercial potential of their early-stage biomedical innovations.

The ETD course teaches commercialization and entrepreneurship concepts in only four weeks; actively forms teams of faculty, graduate students (biomedical and engineering), and medical students for each project; blends in-person with web-based distance learning formats; and is delivered on-site for faculty. In component **D. Translational Endeavors**, we describe how this course and other innovation, commercialization and entrepreneurship educational programs will be made accessible to the entire U-M translational workforce as well as to CTSA hubs across the country using a train-the-trainer method. In this aim, MICHR and FFMI will partner to scale the ETD course to universities and research institutes across the State of Michigan (**Figure H1**). In the first year of the funding period, we will establish links to Wayne State (Detroit), Michigan State University (Lansing), Michigan Technological University (Houghton), and Western Michigan University (Kalamazoo). By year three, we will expand the network to also include members, such as Mercy Health System, Grand Rapids’ Van Andel Institute, Spectrum Health Innovations, Northern Michigan University and Southwest Michigan Innovation Center.

The total potential impact for translating biomedical discoveries into interventions that improve health increases significantly when considering the pipeline of inventions across the affiliated institutions, all of which have biomedical research funding but may have limited internal programs and resources to help in the early-stage commercialization process. With FFMI, MICHR will establish the infrastructure to create a statewide network for biomedical innovation. Using the ETD course as a foundation, we will offer educational programs, skills development, and mentoring that will be complemented by experts local to each spoke. In order to foster statewide engagement in educational opportunities, webinar-based commercialization instruction will also be developed where local and regional partners will contribute to the online program by sharing expert knowledge and resources to help foster commercialization and entrepreneurship throughout the network. Based on a shared need expressed by affiliated spokes for engineering support for early technologies, MICHR will launch a pilot program to connect clinicians with engineers. Technologies in need of engineering support will be identified and matched with student teams made possible, in part, by making projects available to engineering project design courses at affiliated spoke institutions. Mechanisms and agreements to support curriculum-based, student-led product development have already been established at U-M and will serve as a template for other institutions.

*Figure H1. MICHR Statewide Innovation Network. Serving as an innovation hub, MICHR will create an innovation network with 8 other affiliated spoke institutions across the State of Michigan. The yellow dots indicate our first round of partners.*


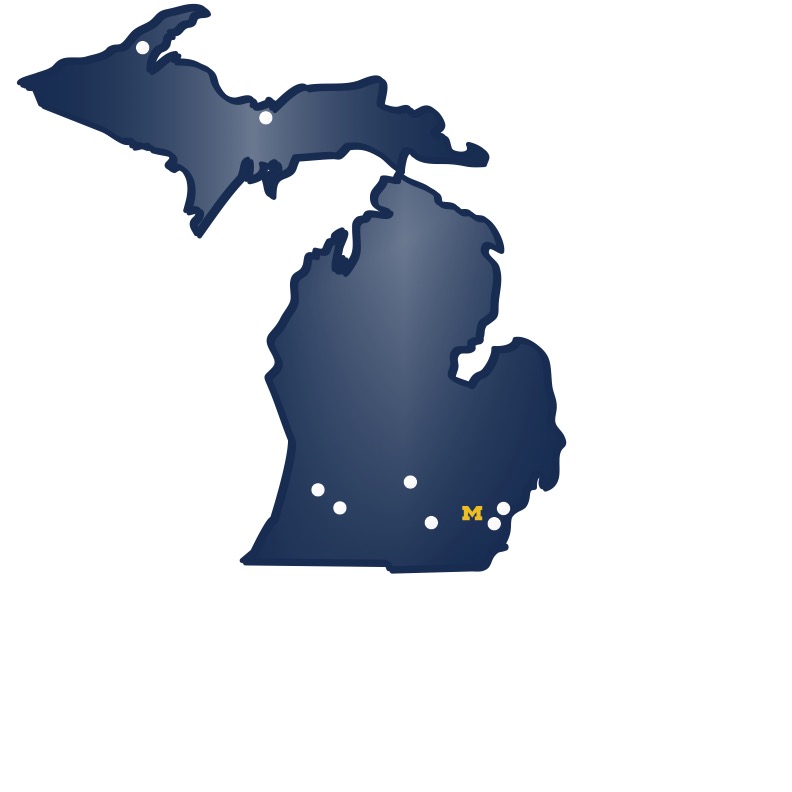


MICHR, with co-facilitation with FFMI, will develop programs and services that not only address individual technology projects but will also cultivate cross-institutional technology development through regional workshops to bring together clinicians, engineers, researchers, and entrepreneurs to identify critical problems and brainstorm potential commercially-relevant solutions. We will facilitate the creation of Technology Commercialization Working Groups, where ideas can be nurtured and further honed. Innovators in a specific domain can come together on a regular basis to share ideas, customer discovery activities, and other best practices. Leveraging the model of the Innovation Navigator Program, faculty champions in specific domains at each affiliated spoke will host working groups joined by entrepreneurs and other business leaders who can provide expert perspectives. Promising projects identified within the network will be developed through institutional and state provided funds. In addition, we will work with and leverage the resources of the respective Technology Transfer Offices at the spoke sites.

**Metrics & Expected Outcomes:** As a result of the innovation network, we anticipate the opportunities for innovation and translation to improve as evidenced by an increase in technology transfer metrics across the State of Michigan as well as an increase in academic-industry collaborations. We expect to see new teams of scientists working together from across the State with increased connections across the spokes of the network. To that end, we will use network analyses to show increased collaborations as well as track proposal submissions and grant awards with investigators from multiple institutions.

**Potential Problems & Alternative Strategies:** To realize the potential of a statewide innovation network will require strong engagement and championship locally at each affiliated spoke, including faculty champions, mentors, and technology miners. MICHR and FFMI will work closely with each institution to help establish these roles and provide training to those individuals as well as provide ongoing coaching and mentorship. Given U-M’s track record with I-Corps™ and the ETD course, we are confident we can deliver commercialization and entrepreneurship training across the network. However, to keep the network engaged, we will have to work in partnership to develop new educational content and offer workshops and events with broad appeal. To achieve this, we will engage the broad network community for input on topics, approaches, and incentives.
